# Strategy for COVID-19 vaccination in India: the country with the second highest population and number of cases

**DOI:** 10.1038/s41541-021-00327-2

**Published:** 2021-04-21

**Authors:** Velayudhan Mohan Kumar, Seithikurippu R. Pandi-Perumal, Ilya Trakht, Sadras Panchatcharam Thyagarajan

**Affiliations:** 1 Kerala Chapter, National Academy of Medical Sciences (India), New Delhi, India; 2 Somnogen Canada Inc., Toronto, ON Canada; 3 Department of Medicine, Columbia University, New York, NY USA; 4 Sri Ramachandra Institute of Higher Education and Research (Deemed University), Porur, Chennai, Tamil Nadu India

**Keywords:** Drug discovery, Immunology, Diseases, Health care

## Abstract

Free vaccination against COVID-19 commenced in India on January 16, 2021, and the government is urging all of its citizens to be immunized, in what is expected to be the largest vaccination program in the world. Out of the eight COVID-19 vaccines that are currently under various stages of clinical trials in India, four were developed in the country. India’s drug regulator has approved restricted emergency use of Covishield (the name employed in India for the Oxford-AstraZeneca vaccine) and Covaxin, the home-grown vaccine produced by Bharat Biotech. Indian manufacturers have stated that they have the capacity to meet the country’s future needs for COVID-19 vaccines. The manpower and cold-chain infrastructure established before the pandemic are sufficient for the initial vaccination of 30 million healthcare workers. The Indian government has taken urgent measures to expand the country’s vaccine manufacturing capacity and has also developed an efficient digital system to address and monitor all the aspects of vaccine administration.

## Introduction

A year has passed since the first case of novel coronavirus infections was detected in China’s Wuhan province. During the initial period of the disease, the efforts were concentrated on preventing and slowing down transmission^1–6^. Global analysis of herd immunity in COVID-19 has shown the urgent need for efficacious COVID-19 vaccines^7^. Currently, the vaccine development efforts have started to come to fruition as some of the leading vaccine candidates have shown positive results in the prevention of clinical disease^8–12^.

Although not mandatory, India with its estimated population of 1380 million (as of 2020) is planning to administer the vaccine to all its citizens who are willing to take it. Importation of vaccines might not be the best option for India due to its large population. According to the International Air Transport Association (IATA), it would require thousands of flights to transport the vaccine from the production sites abroad to the distribution areas.

India, which has a robust vaccine development program, not only plans for domestic manufacture of COVID-19 vaccine but also for its distribution in countries that cannot afford to buy expensive vaccines from the Western world. In India, the data emanating from clinical trials of different vaccines support their eligibility for emergency authorization, even though some of the final details are not available yet. The emphasis now is on the quality control, quality production, and cost control of these vaccines to make them affordable to even the poorest nations in the world.

## COVID-19 vaccine candidates in clinical trials in India

COVID-19 vaccine candidates that are under production and in clinical trials in India are among the leading products internationally. Apart from India’s indigenous COVID-19 vaccines, some local pharmaceutical and biotech companies have signed collaborative agreements with foreign-based vaccine developers. These collaborations range from conducting clinical trials to large-scale manufacturing of vaccines and their distribution. The list of eight vaccine candidates, currently undergoing clinical trials in India, is shown in Table 1 and their technical details are given in Table 2^13^.

**Table 1:** Potential vaccine candidates in India.

Company & collaborating agency	Brand name	Vaccine design	Storage temperature
Serum Institute of India (SII), Pune, India (in collaboration with the University of Oxford, UK, and pharma giant AstraZeneca)	Covishield	Non-replicating chimpanzee adenovirus vaccine vector (ChAdOx1)	2–8 °C
Bharat Biotech Ltd, Hyderabad, India (in collaboration with the National Institute of Virology of ICMR, India)	1. Covaxin^TM^	Inactivated-virus vaccine	2–8 °C
	2. Unnamed		
Cadila Healthcare, /Zydus Cadila, Ahmedabad, India (supported by the Department of Biotechnology, Government of India)	ZyCoV-D	Plasmid DNA vaccine	2–8 °C
Biological E. Limited, Hyderabad, India	1. RBD219-N1	Recombinant RBD protein-based vaccine, with adjuvant CpG 1018	2–8 °C
1. (in collaboration with Dynavax Technologies Corporation and Baylor College of Medicine, USA)	2. Unnamed		
2. Home grown			
Dr. Reddy’s Laboratories, Hyderabad, India (vaccine developed by Gamaleya National Research Institute of Epidemiology and Microbiology, Moscow, Russia)	Sputnik V	Inactivated human adenovirus Ad5 and Ad26 with Spike proteins inserts	−18 °C*
Gennova Biopharmaceuticals Ltd, Pune, India (in collaboration with HDT Biotech Corporation, USA)	HDT-301	mRNA vaccine	2–8 °C

*The Russian company has been testing lyophilisation, turning the liquid vaccine into a dry, white mass that can be stored at normal fridge temperatures of 2–8 °C.

**Table 2:** Technical details of COVID-19 vaccines under clinical trials in India as per ICMR announcement.

Vaccine	Covaxin	RBD219-N1	ZyCoV-D	Covishield	Unnamed	Sputnik V
Clinical Trial Registry India (CTRI) number	CTRI/2020/07/026300	CTRI/2020/11/029032	CTRI/2020/07/026352	CTRI/2020/08/027170	CTRI/2020/11/028976	CTRI/2020/11/029234
Secondary ID	BBIL/BBV152A/2020; version 3.0; date July 7, 2020	BECT062/Covid-19-phase-I&II/CTP-01Ver: 1.1 dated: October 7, 2020	NCOV 1002 version 02 dated: July 2, 2020	ICMR/SII-COVISHIELD version 4.0 dated: October 14, 2020	BBIL/BBV152C/2020, version No: 3.0; date: October 20, 2020	RDI-GCV-001 V3.0 dated: October 19, 2020
Trial type	Interventional	Interventional	Interventional	Interventional	Interventional	Interventional
Study design	Randomized, Parallel Group, Active Controlled Trial	Randomized, Parallel Group Trial	Prospective, Randomized, Adaptive, Phase I/II Clinical Study	Randomized, Parallel Group Trial	Phase 3, Randomized, Double-blind, Placebo-controlled, Multicenter Study	Randomized, Double-Blind, Placebo-Controlled, Parallel-Group, Multi-Centre Phase II/III Adaptive Clinical Trial
Regulatory clearance status from DCGI	Approved/obtained	Approved/obtained	Approved/obtained	Approved/obtained	Approved/obtained	Approved/obtained
Health condition/problem studied	Active immunization for the prevention of SARS-CoV-2 Infection in healthy human volunteers	Active immunization for the prevention of COVID-19 disease in healthy human volunteers	Healthy human volunteers	Healthy human volunteers	Healthy human volunteers	Healthy human volunteers
Intervention/comparator agent	Whole-Virion Inactivated SARS-CoV-2 vaccine (BBV152) with three formulations, BBV152A, BBV152B, and BBV152C. Dose: 0.5 ml, Route of administration:Intramuscular injection, Frequency: two doses at day 0 and day 14.	Biological E’s novel COVID-19 vaccine containing receptor binding domain of SARS-CoV-2 with four formulations (BECOV2D,BECOV2C,BECOV2B and BECOV2A). Dose: 0.5 ml, Route of administration: Intramuscular inj. Frequency: two doses at day 0 and day 28.	Intervention: nCov Vaccine, for Phase I: (1) Arm 1 (1 mg; 0.1 ml) in either of arm with needle; Arm 2 (1 mg) with Pharmajet; Arm 3 (2 mg) with Needle; arm 4 (2 mg) with Pharmajet, in both arm dose.	Intervention - Covishield (SII-ChAdOx1 nCoV-19) - administered as 2 dose schedule on Days 1 and 29 as 0.5 ml dose intramuscularly.	Intervention: BBV152B: 6 μg antigen with Algel-IMDG. Whole-Virion Inactivated SARS-CoV-2 vaccine (BBV152) was be administered as a two dose intramuscular injection 28 days apart.	Intervention: Component I - Recombinant adenovirus serotype 26 particles containing the SARS-CoV-2 protein S gene, in the amount of (1.0+/−0.5)? 10 raise to 11 particles per dose of 0.5 mL.
				Comparator Agent - Oxford/AZ-ChAdOx1 nCoV-19 vaccine. Oxford/AZ-ChAdOx1 nCoV-19 vaccine was administered as 2 dose schedule on days 1 and 29 as 0.5 ml dose.		
					Comparator Agent: Placebo (phosphate-buffered saline with Algel) phosphate-buffered saline with Alum (without antigen) was used as the control. Was administered as a two dose intramuscular injection 28 days apart.	Intervention: Component II - Recombinant adenovirus serotype five particles containing SARS-CoV-2 protein S gene, in the amount of (1.0 +/−0.5)? 10 raise to 11 particles per dose
						of 0.5 mL. Comparator Agent: Placebo Component I - Matching product without Recombinant adenovirus particles containing the SARS-CoV-2 protein S gene. Comparator Agent: Placebo Component II - Matching product without Recombinant adenovirus particles containing the SARS-CoV-2 protein S gene.
			Comparator agent: Placebo in Phase Ii (1) Dose:-0.1 ml in either of arm; Arm 1 (1 mg) with Needle; Arm 2(1 mg) with Pharmajet; Arm 3 (2 mg) with Needle; Arm 4 (2 mg) with Pharmajet, in both arm dose will given.			
			(2) Frequency:-single time at day 0, day 28 and day 56.			
			(3) Route: intradermal			
Blinding/masking	Participant, Investigator, and Outcome Assessor Blinded	Open label	Outcome Assessor Blinded	Participant, Investigator, Outcome Assessor, and Date-entry Operator Blinded	Participant, Investigator, and Outcome Assessor Blinded.	Double-Blind Double Dummy
Sample size	*N* = 1125, with 375 volunteers in the Phase 1 study and 750 volunteers in Phase 2 study (4:1 test and control). Age range: ≥12 to ≤65 years. Both gender	*N* = 360. Age range: 18–65; both gender	*N* = 1048. Age range 18–55; both gender	*N* = 1600. Age range–18–99	*N* = 25800. Age range 18–99. Both gender	*N* = 1600. 100 in phase II and 1500 in phase III. Age range 18–99; both gender
Phase of trial	Phase 1/Phase 2	Phase 1/Phase 2	Phase 1/Phase 2	Phase 2/Phase 3.	Phase 3	Phase 2 and Phase 3
Primary sponsor	Bharat Biotech International Limited	Biological E Limited	Cadila Healthcare Ltd	Serum Institute of India Private Limited	Bharat Biotech International Ltd	Human Vaccine LLC. (Secondary sponsor: Dr Reddys Laboratories Limited)

### Covishield by the Serum Institute of India

Serum Institute of India (SII), Pune, has signed agreements with a few manufacturers such as Oxford-AstraZeneca, Codagenix, and Novavax. It is now producing at a large scale, the Oxford-AstraZeneca Adenovirus vector-based vaccine AZD1222 (which goes under the name “Covishield” in India), and it has stockpiled about 50 million doses^14^. The company will produce 100 million doses per month after January 2021. SII will ramp up its capacity further to produce 2 billion doses per year. Covishield is produced under the “at-risk manufacturing and stockpiling license” from the Drugs Controller General of India (DCGI), and the Indian Council for Medical Research (ICMR). The ICMR funded the clinical trials of the Covishield vaccine developed with the master stock from Oxford-AstraZeneca.

The SII and ICMR have jointly conducted a Phase II/III, observer-blind, randomized, controlled study in healthy adults at 14 centers in India, for comparison of the safety of Covishield (manufactured in India) versus the original Oxford-ChAdOx1 in the prevention of COVID-19 disease. A total of 1600 eligible participants of ≥18 years of age or older were enrolled in the study. Of these, 400 participants were part of the immunogenicity cohort and were randomly assigned in a 3:1 ratio to receive either Covishield or Oxford-ChAdOx1, respectively. The remaining 1200 participants from the safety cohort were randomly assigned in a 3:1 ratio to receive either Covishield or Placebo, respectively. The safety, immunogenicity, and efficacy data of ChAdOx1 administered in two doses containing 5 × 10^10^ viral particles on 23,745 participants aged ≥18 years or older from clinical studies outside India showed the vaccine efficacy to be 70.42%^15^. The safety and immunogenicity data generated from the clinical trial in India was found to be comparable with the data from previous trials conducted outside of India.

The SII has applied to DCGI for permission to do clinical trials in India for Covovax (NVX-CoV2373) developed in partnership with Novavax^16^. They are hopeful of launching the vaccine by June 2021. The US-based pharma claims that their Covid jab was found to be 89.3% effective in a UK trial.

### Covaxin by Bharat Biotech Ltd

India’s first domestic COVID-19 vaccine, Covaxin^TM^, developed and manufactured by Bharat Biotech International Limited, in collaboration with the National Institute of Virology of ICMR, is one of the two vaccines of the company, undergoing clinical trials, and is being stockpiled under an “at-risk manufacturing and stockpiling license”.

Covaxin^TM^ is an inactivated-virus vaccine, developed in Vero cells. The inactivated virus is combined with Alhydroxiquim-II (Algel-IMDG), chemosorbed imidazoquinoline onto aluminum hydroxide gel, as an adjuvant to boost immune response and longer-lasting immunity. This technology is being used under a licensing agreement with Kansas-based ViroVax. The use of the Imidazoquinoline class of adjuvants (TLR7/8 agonists), shifts the T-cell response towards Th1, a T-Helper 1 phenotype (which is considered safer than Th2 responses against SARS-CoV-2) and reduces the risk of immunopathologically mediated enhanced disease^17^.

Bharat Biotech Ltd and ICMR began Phase-III trials of Covaxin^TM^ on November 16, 2020, with 26,000 volunteers across 25 centers in India. According to the company, it is the largest clinical trial in India for a COVID-19 vaccine. The firm has generated safety and immunogenicity data in various animal species such as mice, rats, rabbits, Syrian hamster, and also conducted challenge studies on non-human primates (Rhesus macaques) and hamsters. All these data have been shared by the firm with the drug regulatory body of India. Phase-I and Phase-II clinical trials were conducted on approximately 800 subjects and the results have demonstrated that the vaccine is safe and provides a robust immune response and protection. The Phase-III efficacy trial was initiated in India on 25,800 volunteers and to date, ~22,500 participants have been vaccinated across the country. As per the data available currently, the vaccine is safe. Bharat Biotech has stockpiled 10 million doses of Covaxin and will be ready with another 10 million by February 2021. The company will produce 150 million doses by July–August 2021, and they will be ready with 700 million doses by the end of 2021. The firm is also preparing a protocol to expand the testing of its vaccine in children aged 2–15 years.

### ZyCoV-D by Cadila Healthcare (Zydus Cadila)

Production of another domestic COVID-19 vaccine, ZyCoV-D by Cadila Healthcare, Ahmedabad, based on the new plasmid DNA vaccine technology, is supported by the Department of Biotechnology, Government of India. Vaccines based on plasmid DNA technology are not licensed for public use. Plasmids are used as vectors to directly deliver the DNA encoding the target antigens into the body of the recipient. Sequence encoding for the pathogen’s antigen is engineered into recombinant plasmid DNA. It is used as the vaccine vector so that the vaccine antigens are directly produced by human cells, thus eliciting an immune response. The Phase-I trials of this vaccine began on July 13, 2020, on volunteers of 18–55 years of age. As ZyCoV-D showed promise in a Phase-I study, and the drugmaker Cadila is currently finishing Phase-II trials on over 1000 volunteers across nine sites. This vaccine is administered intradermally.

### COVID vaccine (still unnamed) by Biological E. Limited

Biological E. Limited (BE) has initiated a Phase-I/II clinical trial in India of COVID-19 vaccine (RBD219-N1) produced in collaboration with Dynavax Technologies Corporation and Baylor College of Medicine. BE’s COVID-19 vaccine candidate is based on classical vaccine technology of a protein antigen, SARS-CoV-2 Spike RBD, adsorbed to the adjuvant Alhydrogel (Alum), in combination with another approved adjuvant, CpG 1018. The RBD of the S1 subunit binds to the angiotensin-converting enzyme-2 (ACE2) receptor on the host cell membrane and facilitates virus entry. The results of these clinical trials are expected to be available by February 2021. BE’s Phase-I/II clinical trial will evaluate the safety and immunogenicity of the vaccine candidate at three different doses in about 360 healthy subjects in the age group of 18–65 years. The vaccination schedule consists of two doses (of the same strength) for each study participant, administered via intramuscular injection, 28 days apart.

A locally developed, but still unnamed, COVID-19 vaccine of BE has also been given regulatory approval for clinical trials in India. Details of the drug trials have not yet been disclosed.

### Sputnik V by Dr. Reddy’s Laboratories

Gam-COVID-Vac, trade-named Sputnik V, is a COVID-19 vaccine developed by the Gamaleya National Center of Epidemiology and Microbiology of Moscow, Russia. Sputnik V is a two-vector viral vaccine based on human adenoviruses. Sputnik V uses adenoviruses Ad5 and Ad26^18^. The recombinant adenovirus types 26 and 5 are biotechnology-derived and contain the SARS-CoV-2 S protein cDNA. Both of them are administered into the deltoid muscle. The Ad26-based vaccine is used on the first day and the Ad5 vaccine is used on the 21st day to boost immune responses. Russia’s Sputnik V vaccine stipulates storage at a temperature not higher than −18 °C.

Dr. Reddy’s Laboratories, located in Hyderabad, have received regulatory approval from the DCGI to conduct mid-to-late-stage human trials for Russia’s Sputnik V vaccine in India. Russia’s RDIF-Gamaleya Institute has signed agreements with more than one Indian company for the large-scale manufacture of their Sputnik V vaccine.

### mRNA vaccine (still unnamed) by Gennova Biopharmaceuticals Ltd

The latest COVID-19 vaccine candidate that was granted conditional permission for Phases 1 and 2 of the human clinical trials by DCGI is the mRNA vaccine developed by the Pune-based Gennova Biopharmaceuticals Ltd in collaboration with HDT Biotech Corporation, USA.

## COVID-19 vaccination in India

The government of India has constituted a National Expert Group on Vaccine Administration for COVID-19 (NEGVAC) to provide guidance on all aspects of COVID-19 vaccine administration in India^19^. According to NEGVAC, the COVID-19 vaccine will be offered first to healthcare workers, frontline workers, and to persons above 50 years of age (with first preference for those above 60), followed by persons younger than 50 years of age with associated comorbidities. The government has set up a committee comprising experts from various specialties including oncology, nephrology, pulmonology, and cardiology to define the clinical criteria, based on which people with comorbidities should be prioritized for Covid-19 vaccination. Committee has recommended that anyone with a congenital heart disease that leads to pulmonary arterial hypertension, end-stage kidney disease, or cancers such as lymphoma, leukemia, myeloma, decompensated liver cirrhosis, primary immune deficiency conditions, and sickle cell anemia should be included in the priority. The latest electoral roll for the general election will be used to identify the population aged 45 years or more. The cut-off date for determining the age will be January 1, 2021. There will be a provision for self-registration for vaccination, for those eligible persons who have been missed out from the rolls for one reason or other, after giving some proof of identity. After vaccinating nearly 300 million of the population in the first phase, the remaining population will receive the vaccine based on the disease epidemiology and vaccine availability.

The Government of India has arranged to procure 600 million doses of the COVID-19 vaccine from the manufacturers highlighted above and is negotiating for another billion doses. Covishield, produced by SII, and Covaxin produced by Bharat Biotech Ltd were procured by the government, and are administered initially. Nevertheless, the government may alter its strategy, as and when the other vaccines are cleared for administration after the clinical trials. While obtaining the vaccine is the first requirement, distribution, and vaccination of the huge Indian population presents a significant logistic challenge. On November 24, 2020, Indian Prime Minister Shri Narendra Modi discussed the vaccine distribution strategy with the chief ministers and other representatives of states and Union Territories (UTs). He visited the three leading companies on November 28, 2020 to have first-hand information, and to assure them of full support from the government.

The companies SII, Bharat Biotech, and Pfizer India had applied for “emergency use authorization” of their vaccines. All their applications were reviewed by the expert panel at the Central Drugs Standard Control Organization (CDSCO) for their suitability for vaccination in this country. During the second round of discussions, Covishield, the vaccine candidate from Pune-based SII, was approved for emergency use by the Subject Expert Committee (SEC) of DCGI on January 1, 2021. They have approved the vaccine to be given in two doses 4–12 weeks apart. This time interval is similar to that employed by the UK, and the company is allowed to deploy its vaccines to priority groups, even though a full safety assessment has not been completed. Bharat Biotech was asked to furnish more data demonstrating the efficacy of its candidate, Covaxin. On January 2, 2021, the SEC gave its approval to Bharat Biotech’s Covaxin coronavirus vaccine also for emergency use. These recommendations, along with rollout modalities, were taken up by the DCGI. In a major development, on January 3, 2021, DCGI approved two COVID-19 vaccines for restricted emergency use in the country^20^. Bharat Biotech’s Covid-19 vaccine Covaxin has been recommended for conditional approval (i.e., to be administered under clinical trial mode) by the DCGI based on Phase 3 immunogenicity data for 24,000 volunteers after the first dose, and for 10,000 volunteers after the second dose. Conditional approval of Covaxin was based on incomplete Phase 3 trial data, in the context of a possible emergency, especially infection by mutant strains. On behalf of SEC, Director, All India Institute of Medical Sciences (AIIMS) Dr. Randeep Guleria explained that the Bharat Biotech vaccine will be used in an emergency when there is a sudden increase in cases and need for vaccination. Covaxin can also be used as a backup if questions arise on the efficacy of the SII’s vaccine. According to Dr. Harsh Vardhan, the Health Minister, Covaxin has immunogens (epitopes) from other proteins, in addition to those from Spike proteins. This makes it more likely to work against variants like the N501Y variant (UK variant)^21^. Moreover, Covaxin data showed that it not only produced antibodies in all the participants but it also sensitized CD4 T lymphocytes that impart a durable immune response. The technology used for Covaxin production allows it to target various components of the virus, like the membrane glycoprotein and nucleoprotein, in addition to the spike protein. Managing Director of Bharat Biotech Dr. Krishna Ella said that they will be able to establish Covaxin’s ability to protect against mutant strains of the novel coronavirus, detected in the UK and 30 other countries. Dr. Ella explained that the approval of the SEC of DCGI only means that the firm will no longer require to have a placebo group in its ongoing clinical trial, and will vaccinate people in an open-label format. The safety and efficacy of the drug was to be closely monitored. However, Bharat Biotech announced on March 3, 2021, the results of the third round of clinicals trials showed that Covaxin was 80.7% effective in preventing COVID-19. After going through Covaxin’s Phase 3 trial data, the subject expert committee gave emergency use authorization for this vaccine, and so Covaxin is no longer administered under clinical trial mode.

Pfizer India has reportedly sought more time, but the company’s mRNA vaccine has already been approved, under emergency use conditions, in a number of countries including the USA and UK, and by the World Health Organisation (WHO). Though the extremely low temperature of −70 °C required for storing the Pfizer vaccine poses a big challenge for its delivery in India, the company has hinted at making the necessary arrangements for the same. However, the present laws in India do not normally permit the usage of any vaccine (like the Pfizer vaccine) that has not undergone proper clinical trials in India. Though people above 50 years of age have been prioritized for vaccination by the government, a decision on the administration of Covishield to those above 60 and below 18 had to wait, as clinical trials have not been carried out yet on these age groups of the population. However, the government had relaxed the rules for marketing drugs in India by introducing the “New Drugs and Clinical Trials Rules, 2019”. The need for local clinical trials was also waived in the new rules if the drug is already been approved by any of the DCGI- approved countries, which the DCGI can decide on a case-to-case basis. Approvals by USA, UK, and WHO can be taken into consideration and DCGI may give approval for mRNA vaccine and also for the administration of Covishield and Covaxin to other age groups.

Regional Director, WHO South–East Asia Region, Dr. Poonam Khetrapal Singh, welcomed the first emergency use authorization given to the COVID-19 vaccine. According to her, this decision taken by India will help to intensify and strengthen the fight against the COVID-19 pandemic in the region. The use of the vaccine in prioritized populations, along with the continued implementation of other public health measures and community participation will be important in reducing the impact of COVID-19.

## COVID-19 vaccine distribution: functional cold chain

India has sufficient manufacturing capability for the vaccine (more than 2.4 billion doses annually) and various medical and surgical disposables such as vials, stoppers, syringes, gauze, and alcohol swabs. However, the first bottleneck was the storage and transportation of the vaccines, as this requires very specific temperature regimens. Some of the vaccines under development and production in other parts of the world require storage temperatures as low as −80 °C. Fortunately, the vaccines that India has introduced first for distribution in the country require a storage temperature of 2–8 °C only. The government has been working on measures for the quick and effective distribution of the COVID-19 vaccine. Vaccine manufacturers have started airlifting the vaccines in cold boxes with digital temperature tags to four major depots at Karnal (Haryana), Mumbai, Chennai, and Kolkata, where they are stored in walk-in coolers. From there, planes or insulated vans would transport the vaccines to the designated stores in 37 States/UTs. From these 41 centers, they are further transported to temperature-controlled facilities at the district-level vaccine stores by the State/UT governments. The vaccines are stored in ice-lined refrigerators (ILRs) in districts, from where they are transported to distribution centers in cold boxes and then in ice-packed vaccine carriers to vaccination sites. Real-time remote temperature monitoring of 29,000 cold-chain points is already done through COVID Vaccine Intelligence Network (Co-WIN) vaccine delivery management system, which is a cloud-based digitalized platform. Co-WIN platform was developed by India, but any country can use it. The Indian government will extend assistance for the same.

The initial batches of COVID-19 vaccines are administered through the Universal Immunization Program (UIP) mechanism already operational in India, and it will recruit private cold-chain operators to boost up the capacity. Through UIP, the government is currently, immunizing 26 million children and 30 million pregnant women annually. As UIP has over 26,250 functional cold-chain points at subdistrict or rural level centers (out of its total 28,932 points), vaccines can be stored at facilities not far from the vaccination sites. With the 85,622 cold-chain equipment that UIP has, and the other cold chain infrastructure of the immunization program, the government of India can manage 600 million doses and the private sector can manage 250–300 million doses annually. It could be surmised that with the present capacity, about 400 to 450 million people can be vaccinated in India annually. It indicates that vaccination in India that started from January 2021 would be able to vaccinate only about one-third of the population, by the beginning of 2022, even if it uses the present capacity of the immunization program entirely for COVID-19 vaccination. This is only a theoretical assumption, as India cannot afford to neglect all other vaccination programs under UIP, for the sake of COVID-19. Thus, there is an urgent need to expand the cold-chain infrastructure for storage and transport, as India, the world’s second-most populous nation, moves into the next stage of managing the COVID-19 pandemic. On October 15, 2020 itself, Dr. Harsh Vardhan, the Health Minister of India had directed the states to make a robust plan for vaccine storage and distribution.

The government of India would look for companies, including private ones in each city, which have cold storage facilities and which can take care of distribution, under the regulatory control of the government. Synergistic use of the food cold chain is what the government can use during this time of health emergency. Their facilities would require some minor redesign of storage and transportation. The food cold chain normally has the necessary infrastructure facilities and a complex supply of chain logistics and management.

## Manpower requirement

India’s UIP currently has 55,000 cold-chain staff, and 2.5 million health workers. It will be the health workers, as first-line responders who are getting the vaccination initially. According to government officials, the current healthcare infrastructure may not require additional manpower for administering the vaccine to the healthcare workers. For the second round of vaccination of the priority groups such as the elderly population, persons with comorbidities, pregnant women, and children, a much larger number of trained medical and paramedical staff experienced in vaccine administration will be in place to handle the workload. Understandably, these newly recruited staff will receive the vaccination before they become members of the workforce. Vaccination of people above 60 years and those above 45 with comorbidities have already started from March 1, 2021.

The government of India had asked the states to start training the additional personnel. The orientation of vaccinators through a virtual platform had started on December 5, 2020. The government of India has launched ‘Integrated Govt. Online training’ (iGOT) portal on the Ministry of Human Resources and Development (HRD)’s Digital Infrastructure for Knowledge Sharing (DIKSHA) platform for the capacity building of frontline workers on COVID-19. This platform has training resources that may be accessed by health staff in case they are unable to access the training session or if they want to revisit the training resources. The identified manpower from all the states has been trained in handling the Co-WIN system. COVID-19 vaccine is now introduced only after all training is completed in the district/block/planning unit levels.

Though virtual training methods were used wherever possible, the majority of the training was conducted through the classroom platform. The newer training modalities emphasized “the new normal”, i.e., mitigation of the risk of transmission. For this purpose, 49,604 Medical Officers (in 681 districts) were trained on operational guidelines. The government has already trained 2360 trainers, who would, in turn, train immunization officers, cold-chain officers, IEC (Information, education, and communication) officials, and development partners. As we are reporting, more than 7000 of them have already completed their training, though the number of people required will be several times more than that. More than 18,000 new blocks have been created for vaccination. Trained vaccination teams have already been deployed at 1400 blocks. Each vaccination team will have five members consisting of qualified and trained vaccinators, support staff, and security staff.

As India is planning to have COVID-19 vaccination programs in the urban and rural areas simultaneously, midwives and auxiliary nurse midwives, who have a far greater reach in the interiors and rural areas, were included in the first group of health workers trained in vaccination skills. These trained resources will play a crucial role in the health care of people in rural India. In order to expand a vaccination campaign, the government is planning to engage the allied healthcare workforce including pharmacists and public health workers. Pharmacists may be able to do a better job as a second line of “vaccination warriors” as they have a professional knowledge of maintaining the cold chain and keeping the vaccine intact. It is suggested that the 0.8 million-strong pharmacists in the country could play an important role in this endeavor. At the same time, the existing laws and regulations need to be amended to permit pharmacists to administer vaccines.

The majority of vaccines currently under clinical evaluation need to be administered through the intramuscular route. Later, the vaccinators can be trained adequately to have first-hand experience for administering all modes of injections namely intramuscular, subcutaneous, and intradermal modes. In addition, they should be able to handle different brands of COVID-19 vaccine that will become available in the country in the future. Those vaccines may require a different set of norms for storage and administration. After all this training (e.g., train-the-trainer), the trained personnel can be employed in other places and for other vaccination programs even after this pandemic subsides. They are also trained to monitor and manage common adverse reactions to the injection.

A large number of private clinical laboratories, including diagnostic laboratories, have been established, not only in urban areas but also in the rural areas in India. Most of them have good infrastructural and manpower support. If they become part of the COVID-19 vaccination program, it would be beneficial to both the government and the private clinical laboratories. All these ventures will be executed under strict regulatory control, following standard protocol established by the government agencies, with a standard operating procedure (SOP) to guide the trained workforce. States are augmenting the state helpline 104 (which will be used in addition to 1075) for any vaccine/software-related query. Orientation and capacity building of the call center executives have taken place in the states and UTs.

## Implementation of the program

COVID-19 vaccination, at least in the initial phase, will be totally under government control. High-level coordination at national, state, and district levels have been established for effective cooperation and collaboration among the key departments involved in COVID-19 vaccination. Twenty-three ministries/departments and numerous developmental partners are involved in planning for the COVID-19 vaccine introduction. Their roles have been described in the operational guidelines issued by the Ministry of Health and Family Welfare, Government of India^19^. Co-WIN system will be linked to existing UIP programs and it will meticulously monitor and follow up on the immunized individuals. The Co-WIN system will be used not only to track enlisted beneficiaries but it will also to ensure that only pre-registered beneficiaries will be vaccinated in accordance with the prioritization. Enlisted beneficiaries can select vaccination sites nearest to their home. Autogenerated SMS/email intimations are sent to the beneficiaries, vaccinators, mobilizers, and supervisors about the date, time, and place of the session. To observe the staggered approach, beneficiaries are advised by mobilizers to come to the session as per the staggered time slot to prevent overcrowding at the session site. As per the guidelines issued by the Centre for the COVID-19 inoculation drive, 100 people will be injected in each session per day. People will be monitored for 30 min after administering the shots for any adverse event. On the basis of the initial experience, some vaccination sites have been permitted to work for 24 h every day and the number of people to be injected in each session has been increased up to 200, to speed up the vaccination. Experience gained in the vaccination of the first round would be helpful for the improvement of the second and subsequent cycles.

## Conclusion

The purpose of this perspective was to highlight the overall crux of the vaccine development and vaccination strategies that were implemented during a pandemic in a densely populated country (India). This report can be viewed as a baseline document for future pandemic preparedness, and to effectively tailor and refine the strategies that will help the population at large^22,23^.

India is in a privileged position in producing affordable medical, surgical, and essential generic medicines for the world. It is also well-known that India is the world’s largest manufacturer and worldwide distributor of vaccines. The current COVID-19 pandemic has triggered rapid development, emergency use authorization, and unprecedented collaborative efforts from various stakeholders. Although vaccination might be a cost-effective strategy for survival and a better quality of life for the people as well as for the revival of the economy of India, questions remain. For example, vaccination might not work for some individuals. This, in turn, requires a periodic re-evaluation of the vaccine platforms. Owing to these barriers and gaps in our understanding, the efficacy and safety of COVID-19 vaccination through post-marketing surveillance is of paramount importance and requires long-term follow-up. This should account for both successes and failures, outstanding benefits, and/or its superiority over other types of pharmacological and non-pharmacological treatment regimens. Studies are needed nationally and globally, along with transparent sharing of data and reports among all participating companies, institutions, and nations. This is mandatory for periodic evaluation and re-strategizing COVID-19 management plans. These data analyses can hold the keys to the future effective public health management of COVID-19. India’s experience in immunization for COVID-19 offers tips for strategy preparation, not only for countries with similar economic strength and health facilities but also for the world at large.

## Data Availability

No datasets were generated or analyzed during this study.
